# A machine vision system for automated non-invasive assessment of cell viability via dark field microscopy, wavelet feature selection and classification

**DOI:** 10.1186/1471-2105-9-449

**Published:** 2008-10-21

**Authors:** Ning Wei, Erwin Flaschel, Karl Friehs, Tim Wilhelm Nattkemper

**Affiliations:** 1 Bielefeld University, Faculty of Technology, Fermentation Engineering, P.O.-Box 100131, 33501 Bielefeld, Germany; 2 Bielefeld University, Faculty of Technology, Applied Neuroinformatics Group, P.O.-Box 100131, 33501 Bielefeld, Germany

## Abstract

**Background:**

Cell viability is one of the basic properties indicating the physiological state of the cell, thus, it has long been one of the major considerations in biotechnological applications. Conventional methods for extracting information about cell viability usually need reagents to be applied on the targeted cells. These reagent-based techniques are reliable and versatile, however, some of them might be invasive and even toxic to the target cells. In support of automated noninvasive assessment of cell viability, a machine vision system has been developed.

**Results:**

This system is based on supervised learning technique. It learns from images of certain kinds of cell populations and trains some classifiers. These trained classifiers are then employed to evaluate the images of given cell populations obtained via dark field microscopy. Wavelet decomposition is performed on the cell images. Energy and entropy are computed for each wavelet subimage as features. A feature selection algorithm is implemented to achieve better performance. Correlation between the results from the machine vision system and commonly accepted gold standards becomes stronger if wavelet features are utilized. The best performance is achieved with a selected subset of wavelet features.

**Conclusion:**

The machine vision system based on dark field microscopy in conjugation with supervised machine learning and wavelet feature selection automates the cell viability assessment, and yields comparable results to commonly accepted methods. Wavelet features are found to be suitable to describe the discriminative properties of the live and dead cells in viability classification. According to the analysis, live cells exhibit morphologically more details and are intracellularly more organized than dead ones, which display more homogeneous and diffuse gray values throughout the cells. Feature selection increases the system's performance. The reason lies in the fact that feature selection plays a role of excluding redundant or misleading information that may be contained in the raw data, and leads to better results.

## Background

Discovery of new biological information and knowledge extracted from all kinds of biological entities has been hotspot in recent biomedical researches. These entities have included macromolecules (e.g. DNA, RNA, protein), subcellular structures (e.g., membrane, nucleus, mitochondria), cells, tissues, organs, and so on. Much effort has been made in finding the connections between phenotype and genotype, between function of a biological system (like a cell) and its properties (proteome, transcriptome, metabolome, etc.). Obviously, cell viability is one of the basic properties indicating the physiological state of the cell, thus, has long been one of the major considerations. Recently lots of projects have been carried out on studying mechanisms of cell death [1-4]. In general, viable cells can be distinguished from dead ones according to either the physical properties, like membrane integrity, or their metabolic activities, such as cellular energy capacity, macromolecule synthesis capacity, or hydrolysis of fluorogenic substrates. Conventional methods for extracting information about cell viability usually need reagents to be applied on the targeted cells, and comprehensive reviews of these methods can be found in Ref [5-7]. These reagent-based techniques are reliable and versatile, however, some of them might be invasive and even toxic to the target cells.

Much effort has also been made in developing noninvasive, reagent free methods for measuring cell viability, because the latter are more suitable for on-line or *in situ *monitoring of cells, for instance, in bioreactors. Typical on-line instruments are based on, e.g., capacitance (Aber Instruments Ltd, Aberystwyth, UK), infrared sensing (Finesse LLC, California, USA), and turbidity (Aquasant Messtechnik AG, Bubendorf, Switzerland). Recently an *in situ *dark field microscopy probe for online monitoring of cell density and viability in bioreactors has been proposed [8,9]. With the rapid progress in machine learning and pattern recognition, more and more biological research can be carried out via image-based techniques [10-13]. Wei et al. developed a method to detect cell viability based on evaluation of time series images [14], in which multiple micrographs captured at different time points are needed to extract information for cell classification. Here, a Machine Vision System (MVS) is proposed for automated noninvasive assessment of cell viability. This MVS employs dark field microscopy plus modern image processing. It need not use time series images, but distinguishes live and dead cells by analyzing only micrographs captured at a single time point. In contrast to the system developed by Long et al. [15], which employs iterative training procedures to choose the most representative samples for the decision boundary, our system is focused on selection of the features of cell images that support the best classification of cell viability.

## Results and discussion

**Figure 1:**
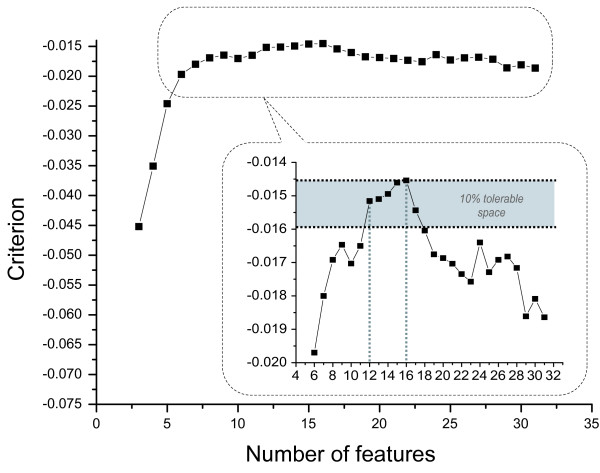
**Feature selection results using Sequential Backward Floating Selection.** The global optimum is found to be the best 16-featured subset. Given a tolerance of 10%, the best 12-featured subset is also acceptable.

The implemented microscope uses a dark field condenser with an numerical aperture (NA) of 0.96 and a 40× objective that has an NA of 0.65. The light source is a Halogen reflector lamp. A CCD camera (XCD-X700, Sony Inc., Tokyo, Japan) is installed to capture the micrographs. This camera is working in visible light range. 10 probes are sampled from the all-live culture and imaged with the CCD camera, and the resulting micrographs are analyzed by the MVS. In the cell detection procedure of the MVS, a reading window of 31 × 31 pixels is used to scan the images, and 466 live cells are recognized in these images. Analogously, 491 dead cells are detected in the images of 10 samples of the all-dead culture. From these, 232 live cells and 247 dead cells are used to generate the training set; while the remaining 234 live cells and 244 dead cells are used to generate the test set. Feature selection is performed and the SVM (Support Vector Machine) classifier with a linear kernel is trained with these datasets. The best subsets of wavelet features are selected with the SBFS algorithm, in which a criterion function is defined in the form of Eq. (10). The selection results are shown in Figure 1. The optimum is found when the number of features is 16, with a criterion value of -0.01454. It is evident that the decline of the criterion within 10% is tolerable (the shadowed region shown in the inset of Figure 1), as a result, the best choice of the feature number should be 12, which leads to a criterion of -0.01516, within the tolerable region. In this 12-featured subset, features 0, 1, 2, 3, 5, 6, 10, 16, 19, 20, 24, 28 are included. When too many features are discarded the criterion declines significantly, especially when the feature number is less than 6.

In order to evaluate the performance of the MVS with cultures of given viability, mixed cultures are prepared. Mixed cultures are obtained by mixing all-live and all-dead cultures at a series of ratios (1:4, 2:3, 3:2, 4:1, 17:3, and 9:1). For each mixture, the reference viability is determined by taking the average of five manual counts based on the FUN 1 stain. The numbers of live and dead cells in these counts and standard deviations are shown in Table 1. As the cell density of the all-live cultures is slightly different from that of the all-dead cultures, the outcome viability is slightly deviated from the nominal value of the mixing ratio. For instance, the nominal viability of the 2:3 (all-live to all-dead) mixture is 0.4, while the actual value is nevertheless about 0.37. The viability determined by the FUN 1 stain is regarded as the gold standard and compared with that by the MVS. For each mixture, five samples are investigated by the MVS and the viability values are averaged.

**Figure 2:**
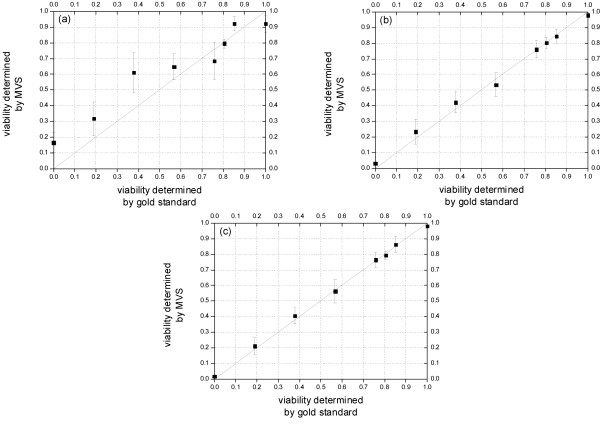
**Performance evaluation of the MVS.** Correlation analysis of the results given by the gold standard and by the MVS (a) using raw image patches; (b) using the complete set of 32 wavelet packet features; (c) using a selected subset of 12 features. It can be concluded that feature extraction and selection have significantly enhanced the system.

The correlation of the results given by the MVS and those by the gold standard is displayed in Figure 2. The total number of the cells in the test sets of all mixed cultures for the MVS is 1702. The system performance is evaluated in three different cases. In Figure 2(a), the training set and test set of the classifier are composed of only raw image patches (namely, without feature generation and selection). In Figure 2(b) the complete set of 32 wavelet features is used. In Figure 2(c) a selected subset of 12 features is used. The effect of feature extraction can be recognized in these figures. Comparing Figure 2(a) with 2(b), it can be seen that the use of wavelet features leads not only to stronger correlation with the gold standard, but also to lower variances. By comparing Figure 2(b) and 2(c), it is clear that discarding 20 features (from 32 to 12 features) does not impair the system's performance. On the contrary, the selected feature set helps not only to increase the accuracy of the measurement, but also to reduce the variance in spite of a slightly increased variance at the viability of 0.85.

**Table 1:** Statistics of the fluorescent reagent based manual counts for the determination of the reference viability of the mixed cultures

MR*	count 1		count 2		count 3		count 4		count 5		ML**(%)	SD***
	#live	#dead	#live	#dead	#live	#dead	#live	#dead	#live	#dead		
1:4	34	163	30	147	24	150	36	144	48	141	18.7	0.044
2:3	65	118	72	116	67	118	80	108	77	127	38.1	0.027
3:2	95	86	93	83	110	65	115	69	101	86	56.9	0.052
4:1	154	39	121	37	120	42	130	52	130	38	75.9	0.032
9:1	143	38	158	37	147	26	131	34	135	39	80.4	0.028
17:3	165	19	130	31	156	22	150	16	142	36	85.6	0.050

* MR: mixture ratio; ** ML: mean percentage of live cells; *** SD: standard deviation

In the course of feature selection, after each backward step a number of forward steps are performed as long as the resulting subsets are better than those previously evaluated at that level. With this so-called floating search mechanism, it will often achieve results close to the optimum, thus, it is valuated as one of the currently best sub-optimal methods for feature selection [16,17]. In this sense, the features most frequently selected at all levels (at different levels, different number of best features are determined at the end of the algorithm) can be considered as carrying the most important information. In our case, features 0, 3, 5, 10, 16, and 19 are the most frequently selected ones. Referring to the definitions in subsection "Wavelet packet feature analysis", these features are associated to subimages (0,0), (0,1), (3,0) and (3,3) of the wavelet packet decomposition. The physical significances of them are given in Table 2 according to the principle of wavelet packet decomposition. These subimages contain the most important discriminative information. It is evident that higher order details in horizontal and diagonal direction (subimage (3,0), (3,3)) and vertical details at a low level (subimage (0, 1)) are critical for classifying live and dead cells.

**Table 2:** The physical significances of the most frequently selected features of the two level complete wavelet packet decomposition (refer to Figure 8)

Feature	Subimage	Physical Significance
0, 16	(0,0)	approximations of approximations (higher order approximations)
5	(3,0)	horizontal details of horizontal details (higher order horizontal details)
3, 19	(0,1)	vertical details of approximations (lower order vertical details)
10	(3,3)	diagonal details of diagonal details (higher order diagonal details)

**Figure 3:**
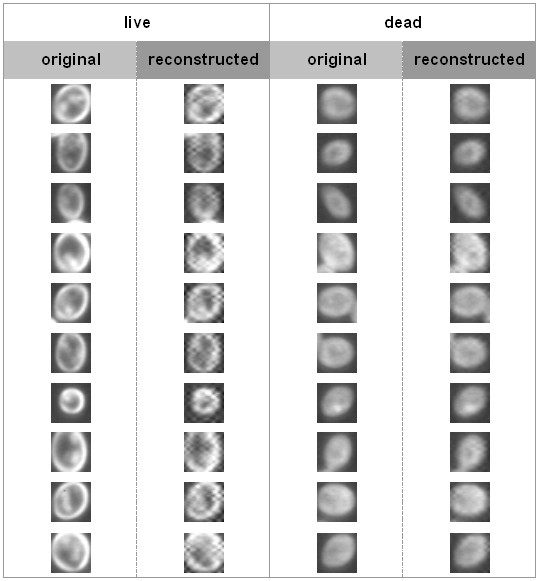
**Comparison of original and reconstructed images of live and dead cells.** Reconstruction is performed through inverse fast wavelet transform after assignment of zeros to the subimages (0,1), (3,0) and (3,3) of the wavelet decomposition. It is clear that discarding of the details information contained in those subimages has much greater influence on live cells than dead cells, which implies that live cells contain more details than dead cells.

This opinion can be supported by a reconstruction of cell images in following steps. Firstly, any of the original cell images is decomposed using FWT. Secondly, a value of zero is assigned to each pixel in subimages (0,1), (3,0), and (3,3). Thirdly, an inverse FWT (IFWT) is used to obtain a reconstructed image, which has lost all the most important discriminative information. The comparison between original and reconstructed images is shown in Figure 3. It can be seen that the reconstructed live cells (column 2) exhibit more "grid effect" than the reconstructed dead cells (column 4), which leads to a greater difference between the reconstructed and original live cells (column 1) than that between reconstructed and original dead cells (column 3). That may serve as evidence for supporting the assumption that live cells contain more detail information than dead ones. Based on Figure 3, it is also clear that with the loss of the information in subimages (0,1), (3,0) and (3,3), which benefits viability classification, live cells are hardly to be distinguished from dead ones.

**Figure 4:**
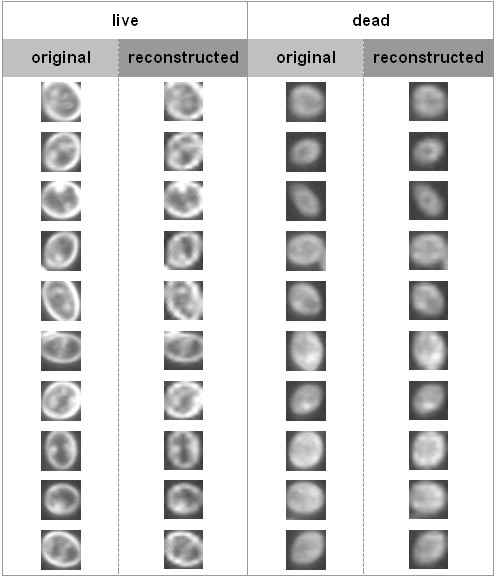
**Comparison of original and reconstructed images of live and dead cells.** Reconstruction is performed through inverse fast wavelet transform after assignment of zero values to the subimages except (0, 0), (0,1), (3,0) and (3,3) of the wavelet decomposition.

Following a similar idea, a value of zero is assigned to each pixel in all the subimages except (0, 0), (0,1), (3,0), and (3,3). This attempt discards all information that is of less significance for distinguishing cell viability. The comparison between the original and reconstructed images is shown in Figure 4. It is clear that the differences between the original and the reconstructed cell images is not so significant as those shown in Figure 3. It is also implied that containing only information in the subimages (0,0), (0,1), (3,0), and (3,3), live cells can still be distinguished from dead ones.

**Figure 5:**
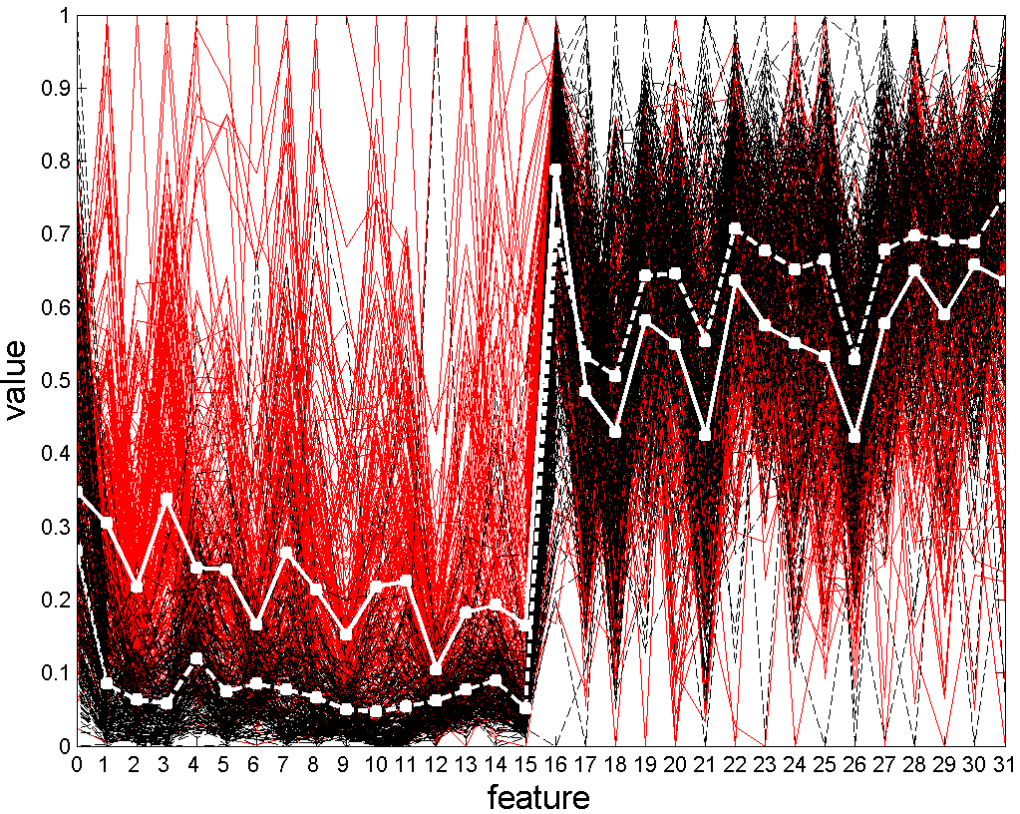
**Feature distribution of live and dead cells.** Thin, gray, solid lines: live cells; thin, black, dashed lines: dead cells; thick, white, solid line: mean feature value over all live cells; thick, white, dashed line: mean feature value over all dead cells. Referring to Figure 8, it is clear that live cells look brighter than dead cells (mean of feature 0), and contain more details (mean of feature 1 ~15). It is also clear that live cells contain more inhomogeneous fine structures than dead cells (mean of feature 17 ~ 31).

For an extended feature analysis, the distribution of the features for live and dead cells from the training set is displayed in a parallel coordinate plot in Figure 5. Each thin, red, solid line represents a live cell, and each thin, black, dashed line represents a dead cell. The mean feature value over all live cells is displayed with a thick, white, solid line, while that over all dead cells is displayed with a thick, white, dashed line. Referring to the definition of features, it is clear that with any energy feature (feature 0 ~15), live cells have a higher mean value. That means, on average live cells look brighter than dead cells (feature 0), and contain more details (feature 1 ~15). It is also clear that with any entropy feature (feature 16 ~31), live cells have a lower mean value (except for feature 16). It implies that live cells contain more inhomogeneous fine structures than dead cells.

In the aforementioned attempts dead cells in both the training sets and test sets have been thermally treated. However, it is yet unclear whether the MVS based measurement is still applicable on cell populations, in which cell death is induced by a different mechanism. In order to validate our methodology, we have tested the MVS on another image set containing cells that have died naturally.

In this scenario the implemented microscope uses the same light source and CCD camera as before, however, it uses a dark field condenser with a numerical aperture (NA) of 0.87 and an 10× objective that has an NA of 0.25. Five probes are sampled from the all-live culture and imaged, and 4131 cell positions are found by the MVS, in which 581 recognized cells are used to generate the positive training set. Analogously, five probes are sampled from the all-dead culture, which is thermally treated, and imaged. 446 out of 1645 recognized cells are selected to generate the negative training set. Besides, the remaining 3550 cells from the all-live culture and 1199 cells from the all-dead culture are added to the test sets for feature selection.

In addition, a naturally grown cell culture *K*_1 _with a known viability of 0.799 (determined by the FUN 1 dye) is also sampled and imaged. In *K*_1 _five probes are taken and in total 2575 cells are recognized (a reading window of 11 × 11 pixels is used to scan the images) as additional test sets for feature selection. The best subsets of wavelet features are selected also with the SBFS algorithm with a criterion function defined in the form of Eq. (11). The optimum is found by SBFS when the number of features is 14. In this best subset, feature 2, 4, 6, 8, 9, 13, 15, 17, 21, 23, 25, 26, 27 and 31 are included. This subset is different from that one determined in the previous scenario, probably because of the different microscopy settings (e.g. different objective magnification factor and different dark field condenser).

In order to evaluate the performance of the MVS, not only all-live and all-dead cultures, but also *K*_1 _and *K*_2 _have been used. The numbers of live and dead cells in these counts and standard deviations of *K*_1 _and *K*_2 _are shown in Table 3.

**Table 3:** Statistics of the fluorescent reagent based manual counts for the determination of the reference viability of the cultures that have been kept for a long time

count #	k_1_			k_2_		
	#live	#dead	%live	#live	#dead	%live
		
1	67	17	79.8	55	14	79.7
2	86	27	76.1	113	27	80.7
3	89	14	86.4	66	13	83.5
4	98	28	77.8	86	21	80.4
5	126	20	86.3	89	14	86.4
6	96	33	74.4	136	27	83.4
7	126	34	78.8	75	21	78.1
8	73	16	82.0	80	7	92.0
9	95	29	76.6	48	11	81.4
10	104	25	80.6	62	13	82.7
						
		mean	79.9		mean	82.8
		SD*	4.1		SD*	4.0

*SD: standard Deviation

The correlation of the results given by the MVS and by the gold standard is displayed in Table 4. It can be seen that not only is MVS reliable for measuring cell viability in all-live (*K*_0_) and all-dead cultures (*K*_3_), but also in *K*_1 _and *K*_2_, which contains cells that are naturally dead.

**Table 4:** Measurement results of the MVS for the cultures *K*_0_, *K*_1_, *K*_2 _and *K*_3_, the viability of which are 1, 0,799, 0,828 and 0, respectively

sample	count 1		cout 2		count 3		count 4		count 5		mean	SD*
	#live	#dead	#live	#dead	#live	#dead	#live	#dead	#live	#dead	%live	
		
*K*_0_	741	26	283	30	310	6	304	33	303	25	93.5	0.036
*K*_1_	492	128	474	100	311	69	430	104	412	55	82.5	0.034
*K*_2_	409	79	401	75	350	70	323	35	255	51	85.0	0.030
*K*_3_	8	273	39	260	9	279	17	253	10	244	5.8	0.042

* SD: Standard Deviation

From the results it can be seen that the MVS is reliable in measuring cell viability. Although it learns from examples of dead cells that were thermally killed (as the negative training set consists of only thermally treated cells), it is able to predict accurately the viability of the cells that have not been thermally treated. Despite of the fact that the selected feature subsets in these two scenarios are different – in this sense, no universal feature subset has been found that is applicable in both cases, it is still evidence that it needs only to train the system with new datasets to have it adapted to a new scenario; however, not a single part of the system framework itself requires any change.

## Conclusion

It has been shown that a machine vision system based on dark field microscopy in conjugation with wavelet feature selection has very good performance in cell viability assessment.

Wavelet features are found to be suitable to describe the discriminative properties of the live and dead cells in viability classification. According to the analysis, live cells exhibit morphologically more details and are intracellularly more organized than dead ones, which display more homogeneous and diffuse gray values throughout the cells.

Feature selection increases the system's performance. The reason lies probably in the fact that feature selection plays a role of excluding redundant or misleading information that may be contained in the raw data, and leads to better results.

Feature selection also reduces the dimensionality of the datasets. That enables the implementation of SVM classifiers with a linear kernel, which are supposed to be unsuitable for high-dimensionality cases. One of the advantages of using linear kernel is that the choice of the proper parameters of a kernel, like the width of the envelop of a Gaussian kernel, can be avoided.

## Methods

### Principle of the MVS

The main idea of the system is to train the MVS with cell samples, the viability of which are known, in order that the MVS learns from the example images some criterion for distinguishing live cells from dead cells just based on their visual appearance. In this learning process, image features are extracted and selected in order to support the classification.

**Figure 6:**
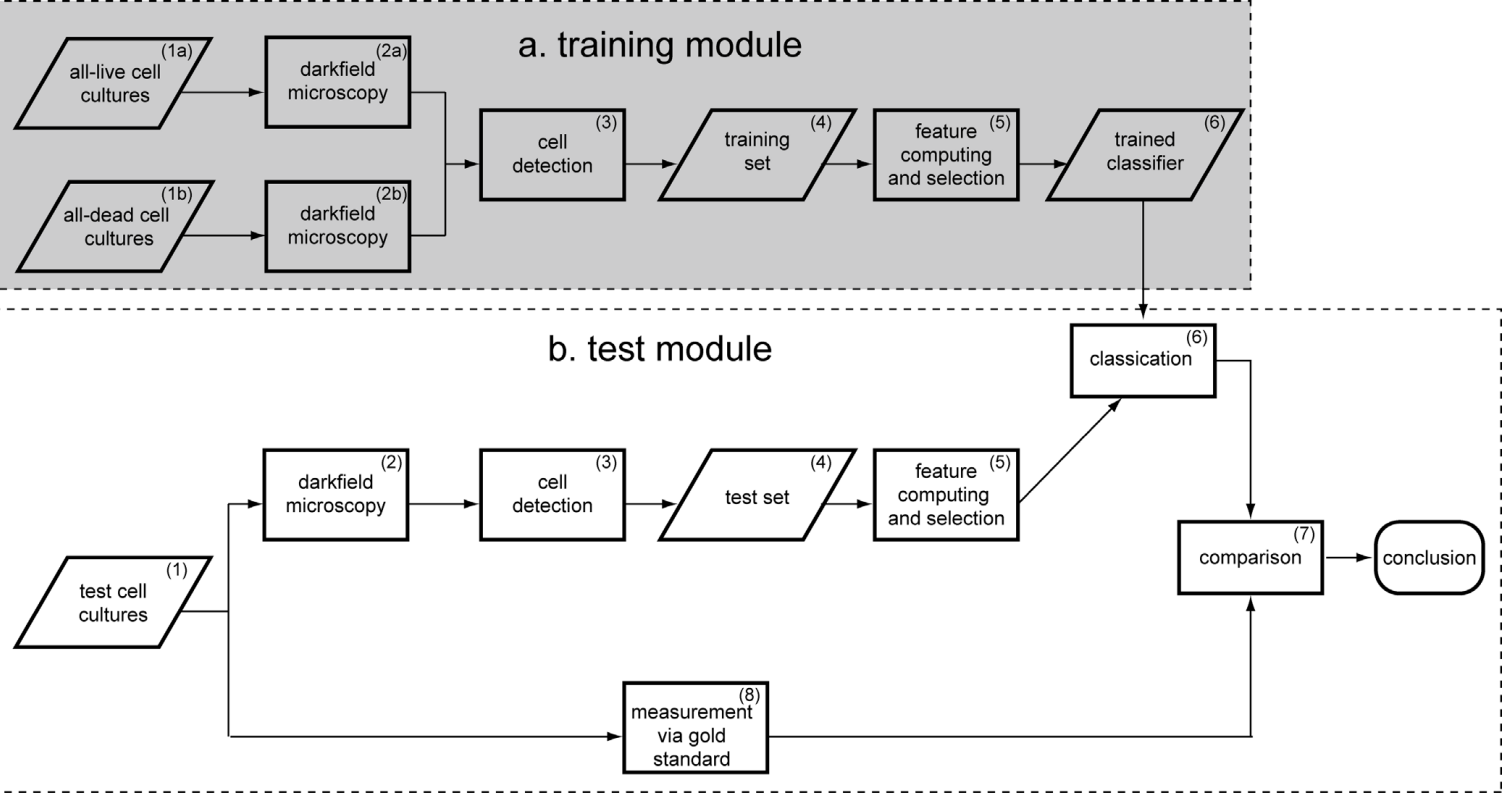
The Machine Vision System composed of the training module (dark shadowed region) and the test module (light shadowed region).

The MVS is composed of two main modules: a training (Figure 6 – a) and a test module (Figure 6 – b). In the training module, two special kinds of cell cultures are used to generate the training dataset. The first kind, the all-live culture (Figure 6 – a (1)) are cell populations in which each cell is alive; while for the second kind, the all-dead cultures (Figure 6 – a (2)), each cell is dead. Micrographs of these cultures are captured with a laboratory microscope under dark field settings, and then a cell detection program as described in [9] is run to find the positions of the cells on the micrographs (Figure 6 – a (3)).

After the stage of cell detection, an image patch of each cell is collected within a window of *N *× *N *pixels around the detected cell centre. These cell image patches compose the training set (Figure 6 – a (4)). Thereafter, features are computed for the *N *× *N *sized image patches prior to performing feature selection (Figure 6 – a (5)), which determines the best subset of these features according to certain criteria so that the best performance can be achieved. A class label *y *= 1 is assigned to the feature vector **x **of each live cell; while a class label *y *= -1 is assigned to that of each dead cell. All of these labelled feature vectors are used to train a classifier (Figure 6 – a (6)) based on Support Vector Machine (SVM) technique [18-20]. Our algorithm is implemented with the SVM-LIGHT (Version date: 02.07.02) [21].

After training the classifier, it can be applied to investigate new cell cultures, in which cell viability is unknown (Figure 6 – b (1)). The test images are processed in the similar way as in the training module: capture of micrographs under dark field microscope, cell detection (Figure 6 – b (2)) and computation of selected feature (Figure 6 – b (3)). The selected subset of features is the same as in the training module. After that, the viability of each tested cell is determined with the SVM classifier (Figure 6 – b (4)). To evaluate the system's performance, the results of different customized cultures are compared with an experimentally derived gold standard (Figure 6 – b (5)) in order to evaluate the system's performance.

### Gold standard of cell viability assessment

In order to evaluate the system's performance, the viability determined by the MVS is compared with that assessed by a commonly used standard method. In this work, a commercial fluorescence probe for live/dead yeast viability evaluation (FUN^® ^1 cell stain, Invitrogen Ltd, Karlsruhe, Germany) is used. With this stain, only live cells are marked clearly with fluorescent intravacuolar structures, while dead cells exhibit extremely bright, diffuse, green-yellow fluorescence. Therefore, the viability of the any cell cultures can be determined easily by manual counting. This viability value is then taken as gold standard.

In this work, the used fluorescence microscope is Nikon Optiphot-2. The protocol of viability assessment with FUN 1 is:

1. Add FUN 1 stain to a yeast suspension at a concentration of 0.5 mM.

2. After incubating yeast for 30 minutes in a dark room, trap 10 *μ*L of the yeast suspension between a microscope slide and coverslip.

3. Examine the stained yeast cells under the fluorescence microscope (excitation: ~450 nm; emission: ~515 nm) and assess manually the ratio of live to dead cells according to the distinguishing intracellular form and color of the fluorescence.

### Strain and medium

Brewer's yeast, *Saccharomyces cerevisiae *(strain Tokay), is chosen to be the target microorganism. In cultivating yeast, a YM medium (glucose: 10 g/L^-1^, peptone: 5 g/L^-1^, yeast extract 3 g/L^-1^, malt extract 3 g/L^-1^, pH 6.2 ± 0.2) is used.

To generate a training set consisting of examples of the live and dead cell images we have prepared all-live and all-dead cell cultures. In order to obtain the all-live cultures, yeast is precultured with the YM medium at 25°C in a 1 L Erlenmeyer flask (filling volume: 0.1 L) on a rotary shaker at the speed of 300 rpm, and harvested in the middle of the exponential growth phase; in order to obtain the all-dead cultures, yeast cells are killed in a water bath at 70°C for 2 hours. It is convenient to obtain a culture with a specific viability by mixing the broth from the all-live and all-dead cultures at a variety of ratios. This kind of cultures is referred to as mixed cultures.

In addition, a naturally grown culture, *K*_1_, which has been kept for 14 days at 25°C in a 1 L Erlenmeyer flask (filling volume: 0.1 L) on a rotary shaker at the speed of 300 rpm with a final viability of 0.799 (determined via the FUN 1 dye), and another naturally grown culture, *K*_2_, which has been kept for 7 days under the same conditions with a final viability of 0.828, have also been prepared.

### Wavelet packet feature analysis

In the MVS, the discrete wavelet transform is performed for feature computation. Wavelet transform as an approach to multi-scale analysis of signals and images has been widely used in image compression, noise removal, texture segmentation, face recognition, medical image processing [22-25], and is explained here only briefly for 1-D signals, which can be readily expanded to 2-D signals, i.e., gray value images.

In Wavelet analysis, a 1D continuous signal *f *(*x*) can be expanded into the following form:

(1) $$f(x)=\sum_{k}W_{\phi ,j_{0}}(k)\phi_{j_{0},k}(x)+\sum_{j=j_{0}}^{\infty }\sum_{k}W_{\psi ,j}(k)\psi_{j,k}(x)$$

where {*φ*_*j*,*k *_(*x*)} and {*Ψ*_*j*,*k *_(*x*)} are sets of scaling functions and wavelet functions, respectively. This series of functions have two parameters: the width, *j*, and the position, *k*:

(2) *φ*_*j*,*k *_(*x*) = 2^*j*/2 ^*φ *(2^*j *^*x *- *k*)

(3) *Ψ*_*j*, *k *_(*x*) = 2^*j*/2 ^*Ψ *(2^*j *^*x *- *k*)

The coefficients $W_{\phi ,j_{0}}(k)$ and *W*_*Ψ*, *j *_(*k*) are determined with following relationships:

(4) $$W_{\phi ,j_{0}}(k)=\int f(x)\phi_{j_{0},k}(x)dx$$

(5) *W*_*Ψ*, *j *_(*k*) = ∫*f *(*x*)*Ψ*_*j*,*k *_(*x*) *dx*

Any of the scaling or wavelet functions can be represented as a weighted sum of scaling functions that have a double frequency:

(6) $$\phi (x)=\sum_{n}h_{\phi }(n)\sqrt{2}\phi (2x-n)$$

(7) $$\psi (x)=\sum_{n}h_{\psi }(n)\sqrt{2}\phi (2x-n)$$

in which *h*_*φ *_and *h*_*Ψ *_are called scaling and wavelet vectors.

If *f *(*x*) is a discretized function (*x *= 0, 1, 2, ..., *M*-1), then Eq. (4) and (5) should be modified to:

(8) $$W_{\phi ,j_{0}}(k)=\frac{1}{M}\sum_{x}f(x)\phi_{j_{0},k}(x)$$

(9) $$W_{\psi ,j}(k)=\frac{1}{M}\sum_{x}f(x)\psi_{j,k}(x)$$

Eq. (8) and (9) are called discrete wavelet transform (DWT), which is performed through operating *f *(*x*) with scaling and wavelet functions. In the fast wavelet transform (FWT) algorithm, relationship between DWT coefficients in adjacent levels is discovered, and the operation is performed with scaling and wavelet vectors (*h*_*φ *_and *h*_*Ψ*_):

*W*_*φ*, *j*-1 _(*n*) = [*h*_*φ *_(-*n*) * *W*_*φ*, *j *_(*n*)]_↓2_

*W*_*Ψ*,*j*-1 _(*n*) = [*h*_*Ψ *_(-*n*) * *W*_*φ*, *j *_(*n*)]_↓2_

where * denotes the convolution operator, ↓2 denotes sub-sampling. It is evident that *h*_*φ *_plays a role as a low-pass filter and *h*_*Ψ *_as a band-pass filter, and the original signals can be split into approximations (*W*_*φ*_) and details (*W*_*Ψ*_). In 2-D cases, such as in classical wavelet decomposition of images, each image is split into approximations and details. The approximations are further split into approximations and details with a 2-D FWT:

$$\begin{array}{l} W_{\phi ,j-1}(m,n)={\left[h_{\phi }(-m)*{\left[h_{\phi }(-n)*W_{\phi ,j}(m,n)\right]}_{↓2(c)}\right]}_{↓2(r)}\\ W_{\psi ,j-1}^{H}(m,n)={\left[h_{\psi }(-m)*{\left[h_{\phi }(-n)*W_{\phi ,j}(m,n)\right]}_{↓2(c)}\right]}_{↓2(r)}\\ W_{\psi ,j-1}^{V}(m,n)={\left[h_{\phi }(-m)*{\left[h_{\psi }(-n)*W_{\phi ,j}(m,n)\right]}_{↓2(c)}\right]}_{↓2(r)}\\ W_{\psi ,j-1}^{D}(m,n)={\left[h_{\psi }(-m)*{\left[h_{\psi }(-n)*W_{\phi ,j}(m,n)\right]}_{↓2(c)}\right]}_{↓2(r)} \end{array}$$

where ↓2(*c*) (↓2(*r*)) denotes sub-sampling along the columns (rows). If the original level of the signals is *J*, then *W*_*φ*, *J *_(*m*, *n*) = *f *(*m*, *n*) is the original image. *W*_*φ*, *J*-*i *_(*m*, *n*) denotes the approximations subimage at level *i*, and $W_{\psi ,J-i}^{t}(m,n)$ denotes the details subimages at level *i *(*t *= *H*, *V*, *D *for horizontal, vertical and diagonal details information).

**Figure 7:**
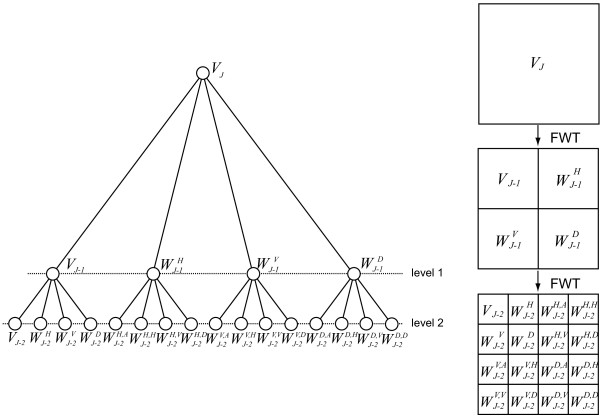
A two level complete wavelet packet decomposition by means of fast wavelet transform.

In a *wavelet packet decomposition*, both the approximations and details are split, which provides richer information for signal analysis. In the proposed scheme, a two levels wavelet packet decomposition is performed, as shown in Figure 7. For simplicity, in this figure *W*_*φ *_and *W*_*Ψ *_are denoted as *V *and *W*, respectively. A Daubechies wavelet with four taps is used for filtering the images. At each level of the decomposition, the frequency space is split into four sub-spaces, which leads to a total of 4^2 ^sub images at level 2, including one approximation and 15 details. Provided that each sub image has a size of *N *× *N *pixels, its energy (*E*) and entropy (*S*) are computed as follows:

$$\begin{array}{c} E=\frac{\sum_{i}\sum_{j}x_{ij}^{2}}{N^{2}}\\ S=-\sum_{i}\sum_{j}p(x_{ij}^{2})\log(p(x_{ij}^{2})) \end{array}$$

in which *x*_*ij *_is the *ij*-th pixel value of the subimage, and *p*(·) denotes the probability of the occurrence of value *x*_*ij*_^2 ^(here the values *x*_*ij*_^2 ^are quantized into 50 bins).

**Figure 8:**
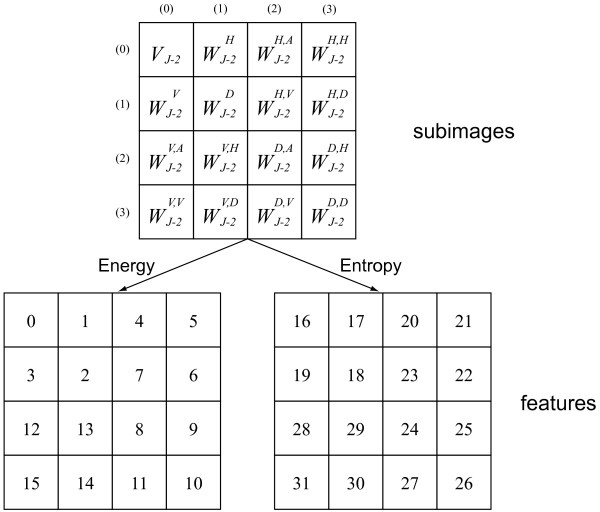
**Layout of the wavelet packet subimages and the features.** For each subimage two features (one energy and one entropy) are computed.

Energy and entropy are computed for all subimages, therefore, in total 32 features can be used for each image. An index is assigned to each feature in accordance with a layout shown in Figure 8. The meaning of the feature can also be determined in this figure. For instance, feature 2 is the energy of the subimage (1,1), namely, $W_{J-2}^{D}$; feature 28 is the entropy of the subimage (0,2), namely, $W_{J-2}^{V,A}$.

### Feature selection

Feature selection has been one of the focuses in pattern recognition because it discovers the subset of features that carries the most discriminative information and abandons those containing more noise than useful information. The advantages of feature selection can be versatile: for instance, reducing dimensionality, enhancing system robustness, increasing recognition rate, and so on.

A large number of algorithms have been proposed for feature selection. Among them, a sequential floating selection algorithm [16,26,27] has been shown to be superior to others in comparative studies. This algorithm can be carried out in two different directions – forward (**S**equential **F**orward **F**loating **S**election, or SFFS) and backward (**S**equential **B**ackward **F**loating **S**election, or SBFS). The source code of these algorithms can be found in [28]. In the former case, the program starts with an empty subset, and searches for the optimal solution by iteratively adding features into the subset; while in the latter case, it starts with a complete set of features, and discards features iteratively. A floating selection process has been applied so that previously added or discarded features still own the chance to be discarded or added, which leads to a higher probability of finding the optimum. If a criterion function *f *($\tilde{X}$) can be determined for any feature subset $\tilde{X}$, then a best subset X* can be found using the SFFS or SBFS algorithm. One essential requirement imposed upon the definition of the criterion function is that the better the subset *S *is, the higher is *f *($W_{\phi ,j_{0}}(k)=\int f(x)\phi_{j_{0},k}(x)dx$).

Feature selection in the MVS is performed based on the performance evaluation of the SVM classifier on determining viability of given cultures. For instance, assume that from the all-live culture *N *samples are taken, and the corresponding viability values determined by the classifier, are denoted as $l_{j}^{\tilde{X}}$ (*j *= 1, 2, ... *M*), $\tilde{X}$ being the present feature subset. From the all-dead culture also *M *samples are taken, and the viability values determined by the MVS are denoted as $d_{j}^{\tilde{X}}$ (*j *= 1, 2, ... *M*).

Suppose the true viability of the all-live culture is *l*_0_, and the true viability of the all-dead culture is *d*_0_, the criterion function *f *with regards to $\tilde{X}$ can be constructed in the following form:

(10) $$f(\tilde{X})=-\frac{1}{M}\sum_{j=1}^{M}{\left(l_{0}-l_{j}^{\tilde{X}}\right)}^{2}-\frac{1}{M}\sum_{j=1}^{M}{\left(d_{0}-d_{j}^{\tilde{X}}\right)}^{2}$$

It is obvious that the higher the criterion function value is, the better is the classifier's performance, and consequently, the better is the feature subset *S*, which satisfies the aforementioned requirement of the SBFS algorithm.

In some cases, the given cultures can also have arbitrary viability. For instance, there are *P *cultures with known viability *v*_1,0_, ..., *v*_*P*,0_, the criterion function can be extended into the following form:

(11) $$f(\tilde{X})=-\frac{1}{M}\sum_{j=1}^{M}{\left(v_{1,0}-v_{1,j}^{\tilde{X}}\right)}^{2}...-\frac{1}{M}\sum_{j=1}^{M}{\left(v_{P,0}-v_{P,j}^{\tilde{X}}\right)}^{2}$$

in which $v_{P,j}^{\tilde{X}}$ is the viability measured by the MVS.

Each *N *× *N*-sized image patch can be depicted as a vector $V_{\omega }\in {\text{R}}^{N^{2}}$. If **V**_*ψ *_is a live cell patch, it is added to the positive training set with a class label "1". Similarly, a dead cell patch is added to the negative training set with a class label "-1". Thus, the whole training set can be interpreted as follows (suppose there are *n*_*train *_training cells):

**Ψ **= {(***V***_*ω*_, ***u***_*ω*_)}, *ω *= 1, 2, ..., *n*_*train*_

The whole training set can also be divided into two subsets **Ψ **= **Ψ**^+ ^∪ **Ψ**^-^, with the positive subset

$$\begin{array}{cc} \Psi^{+}=\left\{\left(V_{\omega^{+}},1\right)\right\}, & \omega^{+}\in \left\{\omega |V_{\omega }\text{ is a live cell patch}\right\} \end{array}$$

and the negative subset

$$\begin{array}{cc} \Psi^{-}=\left\{\left(V_{\omega^{-}},-1\right)\right\}, & \omega^{-}\in \left\{\omega |V_{\omega }\text{ is a dead cell patch}\right\} \end{array}$$

Suppose there are *P *test sets, they are obtained from cultures with known viability *v*_1,0_, ..., *v*_*P*,0_, and from each culture *M *samples are taken. Analogously they can be interpreted as follows:

$$\begin{array}{l} \begin{array}{cc} \Gamma_{l}=\Gamma_{l}{,}_{1}\cup ...\cup \Gamma_{l}{,}_{M}, & l=1,...,P \end{array}\\ \begin{array}{cc} \Gamma_{l}{,}_{j}=\left\{V_{\varphi }\right\},j=1,...,M, & \varphi =1,2,...,n_{test,l,j} \end{array} \end{array}$$

Detailed training procedure is shown as follows:

1. The SBFS algorithm selects a sub feature set $\tilde{X}$.

2. Compute the wavelet features that correspond to $\tilde{X}$ of each input ($V_{\omega^{+}}$, $V_{\omega^{-}}$) in the training set **Ψ**:

$$\begin{array}{cc} V_{\omega^{+}}\to W_{\omega^{+}}^{\tilde{X}}, & V_{\omega^{-}}\to W_{\omega^{-}}^{\tilde{X}} \end{array}$$

3. Train the SVM classifier with ($W_{\omega^{+}}^{\tilde{X}}$, 1) and ($W_{\omega^{-}}^{\tilde{X}}$, -1)

4. Compute the wavelet features that correspond to $\tilde{X}$ of each input (**V**_*φ*_) in each of the test sets **Γ**_*l*,*j*_:

$$V_{\varphi }\to W_{\varphi }^{\tilde{X}}$$

5. Used the trained SVM classifier to assign a class label (+1 for live and -1 for dead) to $W_{\varphi }^{\tilde{X}}$ and thereby determine the viability of each test set ($v_{l,j}^{\tilde{X}}$).

6. Compute the criterion function value with regards to $\tilde{X}$ according to Eq. (11).

7. According to the returned criterion function value, the SBFS algorithm determine whether $\tilde{X}$ is optimal. If not, go to step 1; otherwise, return X* = $\tilde{X}$, and end the program.

## Abbreviations

DWT: Discrete Wavelet Transform; FWT: Fast Wavelet Transform; MVS: Machine Vision System; SBFS: Sequential Backward Floating Selection; SFFS: Sequential Forward Floating Selection; SVM: Support Vector Machine

## Authors' contributions

NW participated in conception, design and test of the system, and drafted the manuscript. TWN contributed to conception and design of the system, and drafted the manuscript. EF and KF participated in design of the system. All authors read and approved the final manuscript.
